# Resuscitation from Chloroform

**Published:** 1874-09

**Authors:** 


Resuscitation from Chloroform.—Dr. Sims, referring to the report in a late number of the *Lancet,* of a resuscitation from excessive narcosis from chloroform, which occurred during an operation for vesico-vaginal fistula, performed by him in Paris, in 1861, related the case in detail, and gave a very graphic and interesting account of the manner in which resuscitation was accomplished. Nelaton, whose patient the lady was, when breathing suddenly ceased, and death from chloroform was imminent, seized the patient's head, and held it downward over the side of the operating table, while another physician present took the legs of the lady and placed them on his shoulders, thus virtually suspending the whole body in air, with the head downward, and a third performed artificial respiration, by compressing the chest. After a considerable interval the patient drew a breath, and when respiration became regular she was again placed upon the table in a recumbent position, but at once respiration ceased again, and the same process of inversion of the body had to be repeated. As soon as respiration became regular she was again placed on the table, and respiration again ceased. The inverted position and artificial respiration were then again employed, and persisted in until the lady became conscious, and asked why she was held in this position. No further relapse occurred, and the operation was concluded without chloroform.

Dr. Sims thinks that syncope during the administration of chloroform is caused by anæmia of the brain, for it is by no means the rule to see patients become cyanotic in the face, but generally pallid, and the inversion of the body, by filling the brain with blood, overcomes the syncope. During the past year he witnessed a second case in the Woman's Hospital, where ether and chloroform mixed were given, syncope occurred, and inversion, with artificial respiration, resuscitated the patient. Dr. Schuppert, of New Orleans, lately reported three cases which he revived in a similar manner, and the method employed in these five cases is certainly worthy of attention and imitation.—*The American Journal of Obstetrics—August.*

